# TEACHING GERONTOLOGY TO SOCIAL WORK STUDENTS: APPLYING THE EXPERIENTIAL LEARNING USING ETHNOGRAPHIC INTERVIEW

**DOI:** 10.1093/geroni/igac059.1530

**Published:** 2022-12-20

**Authors:** Lun Li, Yeonjung Lee

**Affiliations:** MacEwan University, Edmonton, Alberta, Canada; Chung-Ang University, Seoul, Seoul-t'ukpyolsi, Republic of Korea

## Abstract

There is an increasing need for well-trained social workers to support the growing aging population in Canada. Still, concerns arise regarding social work students’ insufficient knowledge and understanding of aging and aging-related issues. This study aims to examine social work students’ experience when experiential learning through ethnographic interview with older adults is applied as a pedagogical approach. This study was conducted based on two cohorts of social work undergraduate students who enrolled in a gerontology course in a Canadian university between 2020 and 2021. Students conducted an ethnographic interview with older adults aged 70 years and older and wrote a reflection paper as an assignment. We did a thematic analysis of eight reflection papers in which consent was obtained from students. We find that students connect aging-related theories/models to various topics discussed during their ethnographic interview, reflect on their personal experiences with aging family members, and show a positive perception of aging and attitude towards working with aging. The findings also suggest the benefit of adopting an approach of experiential learning through the ethnographic interview with older adults to teach gerontology to social work students. We offer recommendations for educators to create opportunities for students, especially from social work or other helping professions who traditionally have shown a lack of interest in working with older adults, to meet and interact with older adults, and to further enhance students’ competencies and interests in the fields of senior care.

